# Advances in Imaging for Atrial Fibrillation Ablation

**DOI:** 10.1155/2011/714864

**Published:** 2011-02-16

**Authors:** Andrew D'Silva, Matthew Wright

**Affiliations:** ^1^Cardiovascular Division, Kings College London BHF Centre, NIHR Biomedical Research Centre at Guy's and St. Thomas' NHS Foundation Trust, London WC2R 2LS, UK; ^2^Department of Cardiology, Rayne Institute, St. Thomas' Hospital, Westminster Bridge Road, London SE1 7EH, UK

## Abstract

Over the last fifteen years, our understanding of the pathophysiology of atrial fibrillation (AF) has paved the way for ablation to be utilized as an effective treatment option. With the aim of gaining more detailed anatomical representation, advances have been made using various imaging modalities, both before and during the ablation procedure, in planning and execution. Options have flourished from procedural fluoroscopy, electroanatomic mapping systems, preprocedural computed tomography (CT), magnetic resonance imaging (MRI), ultrasound, and combinations of these technologies. Exciting work is underway in an effort to allow the electrophysiologist to assess scar formation in real time. One advantage would be to lessen the learning curve for what are very complex procedures. The hope of these developments is to improve the likelihood of a successful ablation procedure and to allow more patients access to this treatment.

## 1. Introduction

Atrial fibrillation (AF) is the commonest cardiac arrhythmia and carries with it significant morbidity and mortality, specifically increasing risk of stroke and heart failure.

With increasing age, AF becomes a more prevalent problem, from 0.5% at 50–59 years to almost 9% at age 80–89 years from US registry data [1]. Figures as high as 17% in those aged 84 years and above have been quoted from European populations [2]. The associated economic burden is considerable and accounts for approximately 1% of the UK's National Health Service (NHS) budget currently, which is expected to rise two-fold in the next 25 years [3].

Management of AF requires individual tailoring to each patient, considering rhythm versus rate control strategies, and managing embolic risk. Since the introduction of the new European Society of Cardiology guidelines on the management of AF, the potential application of catheter ablation has expanded. Catheter ablation is recommended for those patients who are symptomatic and have failed at least one antiarrhythmic medication [4]. 

However, considering patients with minimal or no heart disease, who are symptomatic with paroxysmal AF, the relative safety of the technique in the hands of experienced operators make it suitable for catheter ablation to be considered as an initial therapy in this selected population. Moreover, given the frequent and serious adverse effects associated with long-term amiodarone treatment, in younger patients it is reasonable to consider catheter ablation as an alternative.

Given the complexity and invasive nature of AF ablation, with the small risk of severe complications, there must be sufficient potential benefit to justify the procedure for each individual. Patients with either persistent AF or long-standing persistent AF tend to require extensive and repeated ablation procedures. In addition, in patients with structural heart disease (congestive heart failure, coronary artery disease, and left ventricular hypertrophy) success with catheter ablation is more difficult to achieve. Therefore in these groups, given the lack of a clear benefit-risk ratio, it seems reasonable to recommend that they should be refractory to antiarrhythmic medication before ablation is considered [4]. 

Interestingly, there is evidence that patients with AF-related comorbidity may gain from a primary ablation strategy. In those with heart failure, left atrial ablation conferred significant improvements in ejection fraction and functional end-points such as exercise tolerance [5, 6]. The atrial contribution to the ejection fraction achieved by restoring sinus rhythm in this setting is around 10% [7]. 

## 2. Ablation Strategies

The initial strategy of catheter ablation for atrial fibrillation spawned from the success of the surgical maze procedure, where the atria are cut into electrically disconnected pieces that are too small to sustain AF [8, 9]. From 1995 to 1997, the most common technique employed was the right atrial maze ablation, which enjoyed limited success [9, 10].

Since the demonstration, in 1998, that the majority of patients with paroxysmal AF have the arrhythmia originating from pulmonary vein ectopy [11], catheter ablation has become a far more successful and common intervention. In the US, approximately 50,000 ablations are performed per year, with 60,000 AF ablations in Europe per year. 

Unfortunately, at present, there exists a substantial unmet demand for catheter ablation due to the lack of centres with operators able to perform the procedure. Catheter ablation is technically demanding and operators require a high degree of skill and experience, integrated with an intimate knowledge of anatomy and electrophysiology of the left atrium. This leads to long training times for an electrophysiologist to become fully trained in AF ablation. Even in the hands of the most proficient, success rates for a single ablation procedure for paroxysmal AF are only in the magnitude of 60–85% [12, 13]. For persistent AF, pulmonary vein isolation alone is able to maintain sinus rhythm in only 10–30% of patients [14–16]. 

There are a few possibilities to account for procedure failure. The first is that the pulmonary vein was not truly isolated at the index procedure. The second is that the ablated tissue recovers conduction and the third relies on the development of a new connection. The most likely cause for pulmonary vein reconduction is the first, inadequate lesion delivery at the original procedure. Factors contributing to this include poor catheter stability, inability to achieve transmural lesions, and acute tissue oedema which can cause both temporary isolation and limits power delivery to underlying tissue [17].

In the case of persistent AF, the lower success rates may be attributed to the atria being, more commonly, larger and having more fibrosis [18]. The electrical remodelling that occurs with persistent AF changes the substrate for arrhythmia, so that success with a curative ablation approach, in comparison to paroxysmal AF, is more likely to depend on targeting additional triggers within atrial substrates outside the pulmonary veins [19–21]. As such, often more than one ablation procedure is required to achieve electrical isolation, including the delivery of left atrial linear lesions [22]. Two such useful lines are the left atrial roof line and the lateral mitral isthmus line [23, 24].

Adding complexity to the achievement of successful pulmonary vein isolation is the variable anatomy of the pulmonary veins. Normally, the left atrium has four pulmonary veins draining into it, right and left superior and inferior veins; however, anatomical variants exist. Commonly, these include an additional right middle vein (present in 23% of cases), common ostia (e.g., a left common ostia is seen in 35% of cases) and, less commonly, pulmonary veins that connect to the left atrium by its roof. Successful outcome is determined by the ability to completely electrically isolate these veins [25–27].

Technology has a role here to tackle these factors and improve the success of ablation procedures. Specifically, advances in imaging allow better anatomical localization of left atrial structures to guide lesion delivery and prevent complications. These, although rare, can be devastating. 

When procedures are performed in high-volume centres by high-volume operators, outcomes are better, and major complication rates have been reported in the order of 2-3% [7, 28–33]. However, on a worldwide scale, major complications may be as high as 6% [34]. These may range from complications relating to any vascular access procedure to transient ischaemic attack, stroke, pulmonary vein stenosis, atrio-oesophageal fistula formation, valve damage, cardiac tamponade, and death [4]. 

## 3. Imaging to Help the Electrophysiologist

At present, no imaging modality can serve as a substitute to operator experience and appreciation of each patient's individual anatomy. There is no definitive evidence that any imaging tool used in addition to simple fluoroscopy leads to improved patient outcomes. However, the learning curve to AF ablation is substantial, and advanced imaging modalities may accelerate this process, provide a safety aspect to less experienced operators, and make ablation procedures easier and quicker.

**Figure 1:**
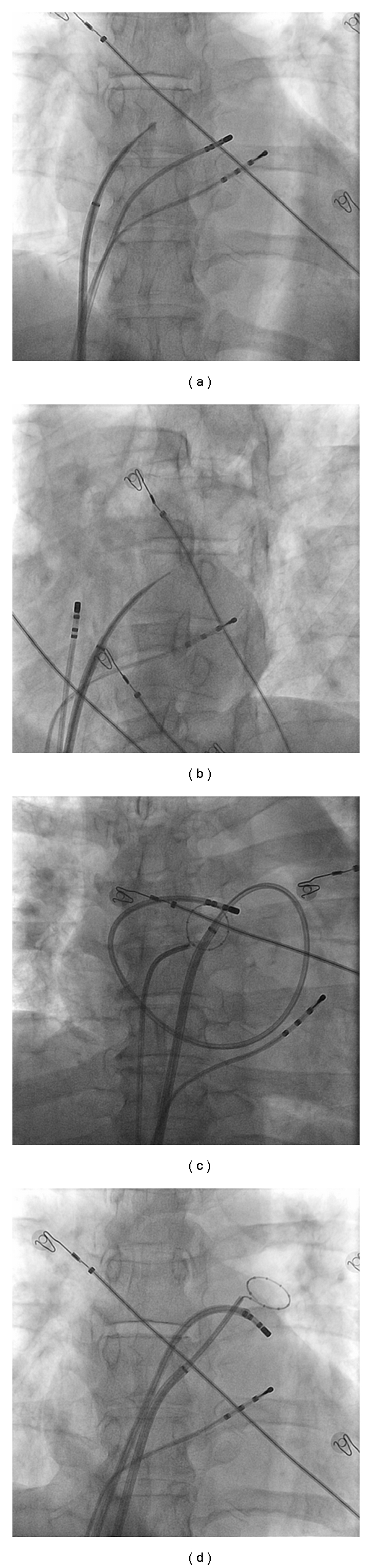
Fluoroscopy as a sole imaging modality. Fluroscopy is still one of the most widely used imaging modalities to guide ablation. Considerable experience is needed to interpret the images and perform a safe procedure. (a) Demonstrates staining of the interatrial septum prior to transeptal puncture (b), at which point a small amount of contrast can just be seen in the left atrium after being injected via the transseptal needle. In (c) a large loop has been made by the ablation catheter, which opposes to the walls of the left atrium. In (d) the ablation catheter is at the ostium of the left superior pulmonary vein with the circular mapping catheter in the left superior vein. The difficulty that fluoroscopy has in visualising soft tissue is apparent.

Every clinical electrophysiology (EP) laboratory is equipped with an X-ray system designed to provide fluoroscopic imaging of the heart. For many years this was the only form of procedural imaging available, occasionally being supplemented with transoesophageal and transthoracic echocardiography in limited cases. Common to all X-ray imaging, although cardiac catheters are well visualised, the soft tissue of myocardium is not. Imaging of the cardiac chambers is indirect through relation to the cardiac silhouette (Figure 1). Even with the limited information gained, selective pulmonary venous angiography is able to help to delineate the pulmonary venous antrum and guide ablation. The walls of the left atrium can be indirectly assessed by bolus injection of contrast, which can be augmented by manoeuvres that minimize atrial emptying such as adenosine or rapid ventricular pacing. Critically, during ablation procedures, fluoroscopy can be used to detect complications from the procedure, such as cardiac tamponade where the excursion of the cardiac silhouette decreases before the development of any clinical compromise in the patient [35]. Inadvertent pericardial puncture and aortic puncture can be identified with characteristic flows of contrast. However, the major disadvantage of using X-ray fluoroscopy as the sole imaging modality is that all images obtained are two-dimensional representations of three-dimensional structures.

## 4. Electroanatomic Imaging Modalities

**Figure 2:**
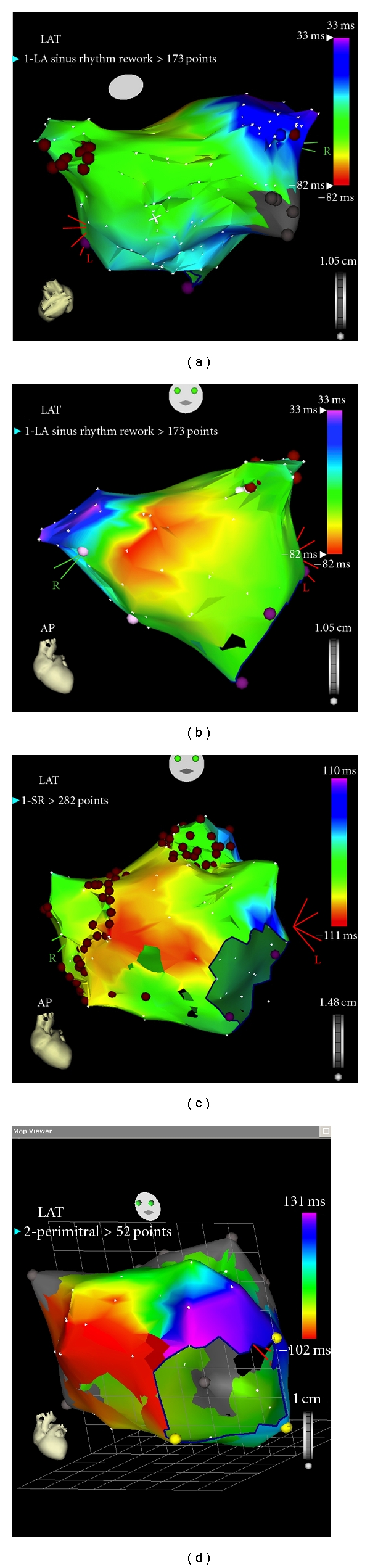
3D electroanatomic imaging. The CARTO system uses small magnets in the tip of the catheter that are able to accurately locate the tip of the catheter within a small magnetic field created by magnets placed under the patient. Using this system, the catheter is moved within the patient's heart to build a 3D shell. Activation timing can be allocated to each point that has been collected to form activation maps. The 3D representation of the left atrium with this early system is still someway from a true representation. (a, b) Demonstrate a posteroanterior and anteroposterior views of a reconstructed left atrium. Ablation lesions have been added during ablation in (c). (d) Is an activation map of the left atrium in a patient with perimitral flutter. The different colours represent isochrones of activation. The entire cycle length can be seen around the mitral annulus.

To combat some of these problems, electroanatomic mapping systems were developed. These systems used magnetic fields or changes in impedance to generate a three-dimensional geometric map of the left atrium. In this way the ablation catheter is localised in three-dimensional space. As with most imaging technologies in their infancy, the maps generated were fairly crude representations of the complex left atrial anatomy (Figure 2). 

Further developments of the imaging systems have led to more accurate representations of the left atrium. The first that came into use for the planning of ablation procedures was preprocedural computed tomography (CT). Although the images obtained of the left atrium were of great value to the operator, unfortunately, CT images could not be displayed concurrently with the fluoroscopic imaging acquired in the electrophysiology lab during the procedure initially. Sophisticated software was used to bridge the gap by development of systems capable of overlaying the segmented CT images of the left atrium directly onto the live fluoroscopy screen [36] or into a 3D electroanatomic mapping system [37–42]. For the electrophysiologist, this brought the beginning of 3D representation of individual patient's cardiac anatomy into the procedural lab, displayed over real-time fluoroscopy. A secondary benefit was that extracardiac structures could also be visualized, such as the oesophagus. When ablating in the posterior wall of the left atrium, one of the most feared and lethal complications that can occur is the formation of an oesophageal fistula [43–45]. By displaying the relationship of the oesophagus to the left atrium, adjustments can be made to the energy delivered through ablation lesions, aiming to minimize this risk.

This integrated system requires manual registration of the segmented left atrium to the patient with various methods available to achieve this. Despite this adjustment, images are remarkably accurate to within 0.3 mm [46]. However, registration of the 3D images is sometimes difficult due to changes that have occurred between the CT scan taking place and the ablation procedure. This underlies one of the most important limitations of this imaging modality. The usual lag time between CT scan and ablation procedure is a matter of days. During this time the patient's rhythm may have changed, the volume status of the patient is likely to be different, and, most importantly, the patient's position on the procedure table is different to that in the CT scanner. These changes may affect the left atrium; however, it is not known whether they produce clinically relevant differences.

**Figure 3:**
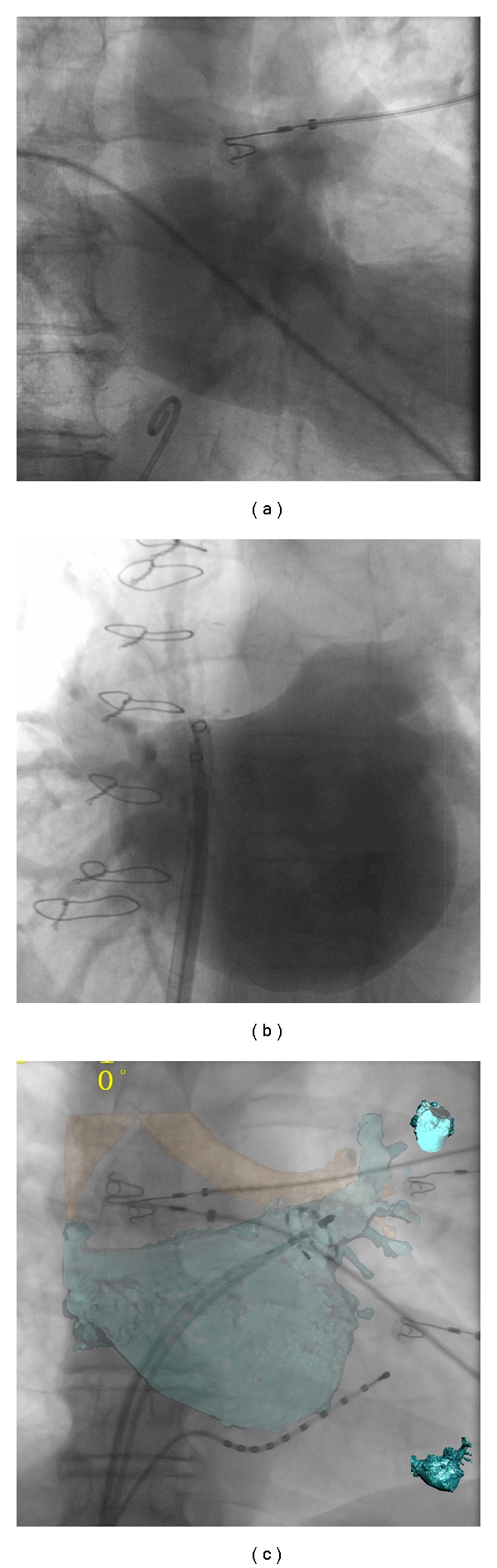
Rotational angiography. A tremendous improvement on fluoroscopy was the development of rotational angiography. Here the C-arm used for fluoroscopy rapidly rotates around the patient while contrast opacifies the left atrium. ((a) Contrast injected in the right atrium and then a delay of 8 seconds until the LA opacifies, (b) direct injection into the left atrium), following segmentation of the resulting dataset, which is semiautomatic; a 3D anatomical accurate shell can then be superimposed on the live fluoroscopy (c) to aid with ablation, or exported to other mapping systems.

Superseding CT imaging, with the aim of overcoming these difficulties, rotational angiography was developed [25, 26, 47–50]. One major advantage is that this modality utilizes the fluoroscopy system that is routinely available in the electrophysiology lab. With rotational angiography, the left atrium is isocentred and opacified with contrast, which can be achieved in a variety of ways [25, 47, 51], then the C-arm rotates around the patient in a 240° arc over 4 seconds. The data set created is read by the electrophysiology system in the same way as a preprocedural CT scan. As such, imaging is performed during the procedure and displayed on the live fluoroscopy screen (Figure 3).

Registration accuracy is not a concern; this can be checked rapidly by placing a catheter within the superior pulmonary veins and looking for the drop off between the pulmonary vein ostium and the left atrial body. If the patient moves during the procedure, the anatomy can be re-registered quickly, using the trachea as an anatomical landmark [52]. Another advantage of rotational angiography over CT is that the radiation dose to the patient is less.

The operator is able to see the exact position of critical structures including the ostia of the pulmonary veins, any anomalous pulmonary veins, and the ridge between the left atrial appendage and the left superior vein. This has the potential to improve the success of pulmonary vein isolation through accurate anatomical planning and also avoid pulmonary vein stenosis, which occurs in around 1–3% of cases [53], when ablation is performed inside the pulmonary vein.

These imaging tools have yet to translate into clear improvement in patient outcomes [54, 55]. In one retrospective study, there was a suggestion that 3D electroanatomical systems with integrated CT imaging led to better procedural outcomes [37]. The same group later published a prospective randomised trial, which did not demonstrate any difference in patient outcomes when CT integration was employed [41]. As the authors concluded, procedural success is dependent on the successful isolation of pulmonary veins, regardless of the technology used to achieve it. This has further been backed up by data on patients undergoing lung transplantation. In patients receiving double lung transplantation (which results in “isolation” of all pulmonary veins) virtually no patients (0.5%) went on to have AF compared to 30% of similar patients undergoing either single lung transplantation or thoracic surgery [56].

Comparing Carto XP to rotational angiography, a two-centre study, with over 90 patients, demonstrated equivalent results in procedural success and fluoroscopy times. A further study by the same group did not demonstrate any differences in procedural or clinical outcome when comparing patients who had the procedure using rotational angiography or a 3D electroanatomic imaging system [52]. 

## 5. Lesion Formation

Despite these tremendous advances in imaging, there is one important area that has not seen comparable growth. This is in the assessment of tissue contact, which is a key determinant of successful lesion development [57].

Currently, systems are being developed to assess and control tissue contact, including robotic [58–61] or magnetic catheter navigations [62–64] and sensors in the catheter tip detecting force [65, 66].

However, at present, forms of lesion assessment are indirect, using changes in tissue impedance, diminution of local electrogram voltage and assessment of contact force.

Magnetic Resonance (MR) imaging shows promise in its ability to visualize scar formation in the left atrium [67–72]. Currently in its infancy for studying the left atrium, interesting reports have emerged in its use following ablation procedures. In a case report, a gap identified in a linear lesion seen on preprocedural MR was found to be responsible for an atrial tachycardia. Ablation at this gap, achieving physiological block of the line, resulted in termination of the arrhythmia [73].

Cardiac MRI has been used to investigate the correlation between points of energy delivery and scar formation. One study showed that 20% of sites where energy was thought to have been delivered had no evidence of scar formation [74]. Furthermore, another study demonstrated that oedema formation might contribute to pulmonary vein isolation acutely. Therefore, on resolution of oedema, the possibility of pulmonary vein reconduction arises [72].

**Figure 4:**
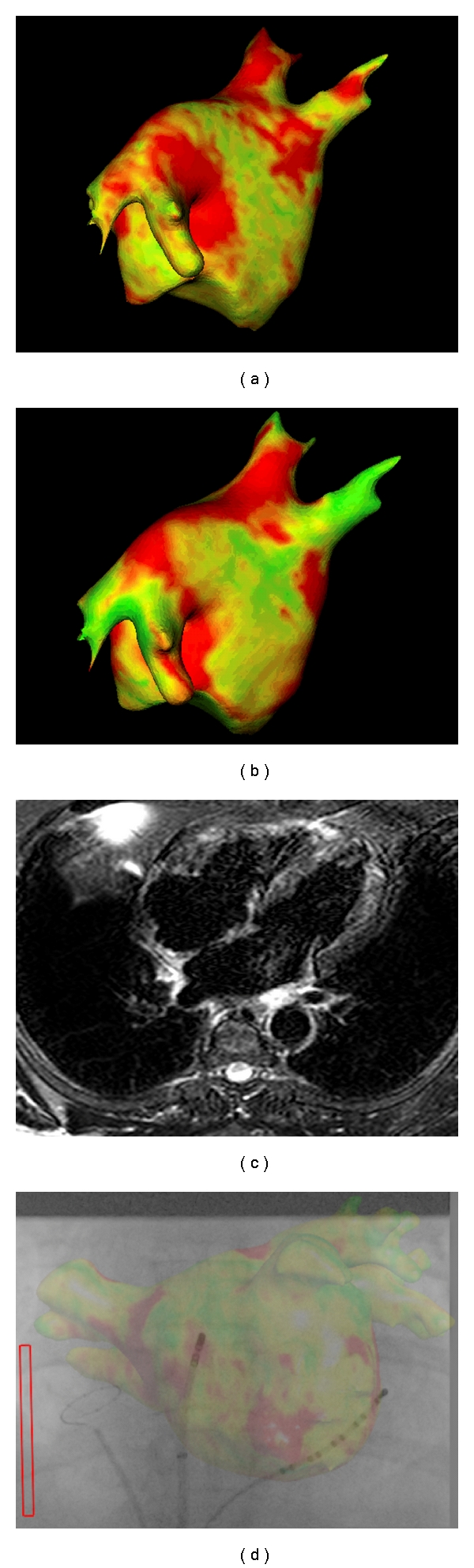
Left atrial magnetic resonance imaging. Postprocedural imaging of the left atrium with delayed enhancement ((a, b)) and T2 weighted (c) to assess left atrial scar and oedema, respectively. The raw image is processed to obtain a 3D representation of the anatomy, with colour-coded representation, which is thought to represent scar formation and oedema. The images may help guide ablation (d). Images courtesy of Ben Knowles and Kawal Rhode, Kings College London and St. Thomas' Hospital.

At present, the clinical information provided by MR on lesion formation is only available following the procedure (Figure 4). In the future, MR imaging may allow real-time imaging of the catheter; however, real-time lesion assessment has not yet been developed [75]. Real-time assessment of the ablation lesion is currently regarded as a holy grail in electrophysiology. Recent work in a sheep model has demonstrated that ultrasound, integrated into an ablation catheter, was able to detect changes in tissue structure that correlate with tissue necrosis [76]. This novel integrated ultrasound catheter was tested in an in vivo sheep model, using both atrial and ventricular lesions. Impressively, for lesions up to 5 mm in depth, accurate real-time myocardial imaging could be used to differentiate between tissue necrosis and viable tissue. Although this shows promise, further work with this technology is required in clinical trials. 

## 6. Conclusions

**Figure 5:**
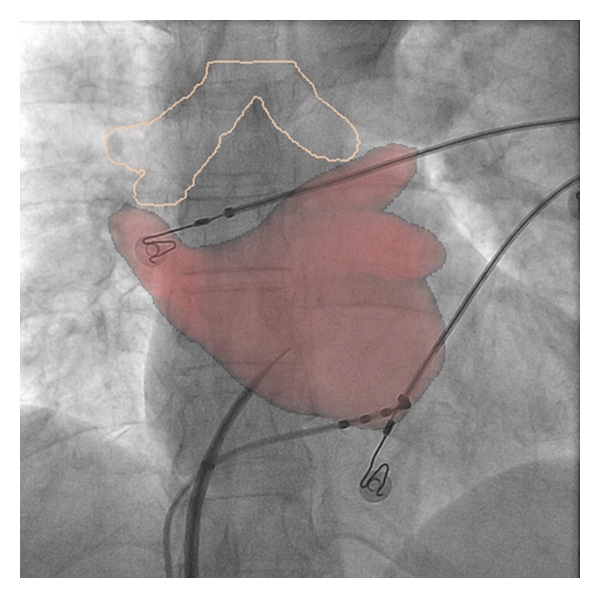
Real-time respiratory-gated motion compensation. A system is being developed whereby the 3D shell of the left atrium (either from CT/MR or rotational angiography) moves during the abaltion in response to respiratory motion. This example demonstrates how the trachea is automatically tracked. Image courtesy of Kawal Rhode, Kings College London and St. Thomas' Hospital.

Over recent years, technological advances in the field have been astounding; the EP lab of the future has even more possibilities. Currently overlaid 3D images are static; however, in the future, real-time visualisation of cardiac contraction and diaphragmatic movement (Figure 5) holds the possibility of more accurate catheter placement and avoiding inadvertent ablation within the pulmonary veins. New information from MR images relaying anatomical areas of fibrosis could be projected onto the 3D lab images. 

Advances in imaging provide much greater anatomical detail of myocardium, which is only half of the story. Electrophysiology is the integration of discrete anatomical structures with discrete electrical properties. Work on software prototypes is currently underway, which will allow electrical data from the EP recording system to be displayed in colour-coded format on the 3D overlay [77]. This could allow for dominant frequency mapping and wavelet analysis in AF in an effort to localise areas critical to the AF process, as well as demonstrating complete conduction block across linear lesions with ease [77].

In summary, imaging tools in current use give the electrophysiologist information that could be obtained by using simple fluoroscopy and possessing considerable operator experience. The success of an AF ablation procedure is crucially dependent on pulmonary vein isolation; it is the accurate understanding of each patient's anatomy that ensures this. Detailed anatomical imaging affords the operator an insight into the complex anatomy of the left atrium and makes the goal more readily achievable, although there is a lack of evidence translating this into improved patient outcomes. Assessments of tissue contact and lesion development remain outside the grasp of the practicing electrophysiologist; however, this may change in years to come. Ultimately, these technologies need to demonstrate proven benefit to patients in procedural success and reducing complications, when compared to current practice.
